# miR-214/199a/199a* cluster levels predict poor survival in hepatocellular carcinoma through interference with cell-cycle regulators

**DOI:** 10.18632/oncotarget.6137

**Published:** 2015-10-19

**Authors:** Peipei Wang, Song Chen, He Fang, Xiaojuan Wu, Dabiao Chen, Liang Peng, Zhiliang Gao, Chan Xie

**Affiliations:** ^1^ Department of Infectious Diseases, The Third Affiliated Hospital of Sun Yat-sen University, Guangzhou, Guangdong Province, China; ^2^ Key Laboratory of Tropical Disease Control, Ministry of Education, Sun Yat-sen University, Guangzhou, Guangdong Province, China; ^3^ Department of Radiology, Guangzhou Red Cross Hospital/The Fourth Affiliated Hospital of Jinan University Medical College, Guangzhou, Guangdong Province, China; ^4^ Department of Burn Surgery, Changhai Hospital, Second Military Medical University, Shanghai, China

**Keywords:** microRNA, human hepatocellular carcinoma, cell cycle

## Abstract

**Aims:**

To identify the clinical and functional association of miR-214/199a/199a* cluster in human hepatocellular carcinoma (HCC) and to clarify the mechanism of miR-214.

**Methods:**

Kaplan-Meier and Cox proportional regression analyses were used to determine the association of miR-214/199a/199a* cluster levels with the survival of HCC patients. The role of miR-214 in regulating HCC cell proliferation was studied with miR-214 mimics/inhibitor-treated cells. Furthermore, the inhibition effect of miR-214 on E2F2, cyclin-dependent kinase (CDK) 3 and CDK6 expression was assessed in HCC cell lines with miR-214 mimics/inhibitors to increase/decrease miR-214 expression. Direct binding of miR-214 to the 3′-untranslated regions of E2F2, CDK3, and CDK6 was verified by dual-luciferase reporter assay.

**Results:**

In analyzing HCC clinical specimens and cell lines, we discovered a uniform decrease in miR-214/199a/199a* expression in comparison with noncancerous tissue or normal liver epithelial cell lines. Higher miR-214 levels were related with improved patient survival. Overexpression of miR-214 in HCC cells inhibited proliferation by inducing G1-S checkpoint arrest. Conversely, RNA interference–mediated silencing of miR-214 promoted cell-cycle progression and accelerated the proliferation of HCC cells. E2F2, CDK3 and CDK6 were each directly targeted for inhibition by miR-214, and restoring their expression reversed miR-214 inhibition of cell-cycle progression. The relationship between expression of miR-214 and its targets was confirmed in HCC tumor xenografts and clinical specimens.

**Conclusions:**

Our results demonstrate that miR-214 has tumor-suppressive activity in HCC through inhibition of E2F2, CDK3 and CDK6.

## INTRODUCTION

Primary human hepatocellular carcinoma (HCC) remains a major public health threat and the third leading cause of cancer death [1]. HCC carcinogenesis and progression involves aberrant changes in multiple molecular pathways, including genetic as well as epigenetic factors [2, 3]. While most studies on HCC have focused on the malignant transformation or proliferation promotion of specific oncogenes, the cross-talk between multiple genes has largely been ignored. Hence, it is of great clinical value to investigate the cooperative effect of multiple genes involved in HCC at the same time, and develop effective therapeutic targets for achieving better prognosis of the disease.

One of the hallmarks of cancer is uncontrolled cell proliferation, leading to malignant tumor development. Dysregulation of cell-cycle progression, such as evasion of multiple cell-cycle checkpoints, can be caused by abnormal activation of two key classes of regulatory molecules, cyclins and cyclin-dependent kinases (CDK) [4]. The cell cycle is activated by CDK6 early in G1 phase through interactions with cyclins D1 [5], D2 and D3. The Cyclin D-CDK6 enzymatic complex, together with Cyclin E/CDK2 phosphorylates the protein retinoblastoma (Rb) which releases its binding partner E2F, a transcriptional activator, which in turn activates DNA replication. The CDK6 complex acts as a switch that commits cells to division in response to external signals, such as mitogens and growth factors [6, 7]. The complex therefore exhibits strong proliferative activity and has been demonstrated as a mechanism for the pathogenesis of HCC [8, 9]. Although distinct cyclins and CDKs play crucial roles in the development and progression of HCC, the effect is often overridden by negative regulators [10–12]. Understanding the interactions between multiple cell cycle regulating genes involved in HCC may lead to more effective therapeutic strategies.

microRNAs (miRNAs) which suppress multiple genes at the same time can provide new clues for the development of targeted therapies against HCC. As a single miRNA can inhibit multiple mRNAs and synergistically suppress various regulators that are cooperatively involved in cell-cycle regulation, identification of such miRNAs might help us to develop targeted therapies against HCC carcinogenesis. Notably, miR-214 was of particular interest as our informatics analysis predicted potential binding sites for miR-214 in the 3-untranslated regions (3′-UTR) of E2F2, CDK3, and CDK6, 3 key regulators of the cell cycle. In this study, we show that miR-214 causes cell-cycle arrest by inhibiting E2F2, CDK3, and CDK6. Importantly, miR-214 expression in HCC cells is markedly reduced, resulting in unrestricted cell-cycle progression and consequently increased cell proliferation. Moreover, miR-214 down-regulation correlates with poor prognosis of patients with HCC, suggesting that miR-214 might act as a tumor-suppressor miRNA in the development and progression of HCC.

## RESULTS

### Reduced expression of miR-214/199a/199a* correlates with HCC progression and poor prognosis

**Figure 1:**
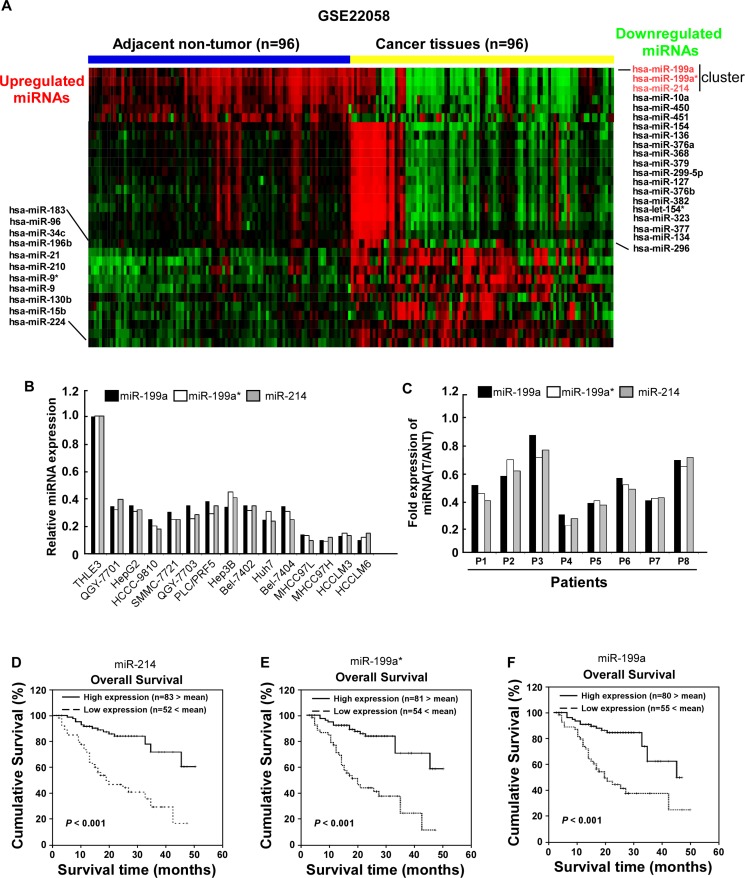
Downregulation of miR-214/199a/199a* in HCC is correlated with poor patient survival **(A)** expression profiling of miRNAs in 96 primary HCC tissues and 96 adjacent non-tumor tissues (NCBI/GEO/GSE22058) **(B)** miR-214/199a/199a* expression was analyzed in immortalized human hepatocyte epithelial cell line THLE3, and indicated human HCC cell lines using qRT-PCR. **(C)** Relative expression of miR-214/199a/199a* in 8 pairs of HCC tumor tissues (T) compared to their corresponding adjacent noncancerous tissues(ANT). Kaplan–Meier correlation analysis between miR-214 **(D)** miR-199a* **(E)** and miR-199a **(F)** levels and overall survival of patients with HCC with high and low miR-214 expression.

By analyzing published miRNA expression profiles obtained from 96 primary HCC tissues and adjacent non-tumor tissues (NCBI/GEO/GSE22058), miR-214/199a/199a* was identified to be significantly downregulated in HCC tissues compared with adjacent non-tumor tissues (Figure 1A). Furthermore, real-time PCR analysis revealed that miR-214/199a/199a* was downregulated in all 14 tested HCC cell lines compared with the immortalized normal liver epithelial cell line THLE3 (Figure 1B) and in 8 HCC samples compared with the matched adjacent nontumor tissues (Figure 1C). Collectively, these results indicate that miR-214/199a/199a* expression is reduced in HCC. To evaluate whether downregulation of miR-214/199a/199a* correlates with clinical HCC progression, we examined miR-214/199a/199a* expression in 135 archived human HCC specimens. Patients with lower miR-214/199a/199a* expression had a shorter survival time, whereas patients with higher miR-214/199a/199a* expression had a longer survival time (*P* < 0.001; Figure 1D–1F). We used qRT-PCR assay to confirm the expression of miR-214/199a/199a* that were selected from the previous step from an independent cohort of 16 serum samples which including 8 HBV related cirrhosis and 8 HCC. miR-214/199a/199a* had significantly different expression levels between the HCC and control cirrhosis groups (data not show). But not as in HCC tissue, miR-199a was up-regulated in HCC serum. While miR-214 was down-regulated in HCC serum as same as in HCC tissue. Importantly, statistical analyses revealed that miR-214 expression inversely correlated with TNM classification (*P* = 0.019) in patients with HCC (Table 1). Univariate and multivariate analyses revealed that clinical stage and miR-214 expression were each recognized as independent prognostic factors in HCC (Table 2). Thus, low miR-214 expression seems to be a risk factor predicting poor survival (Table 3), suggesting that lower expression of miR-214 contributes to HCC pathogenesis and might represent a prognostic biomarker for human HCC.

**Table 1:** Correlation between miR-214 expression and clinicopathologic characteristics of liver cancer patients

Characteristic	miR-214		Chi-square test
	High	Low	*P* value
Age (> 50 versus ≤ 50 years)	32/50	24/29	*0.471*
Sex (M versus F)	72/10	50/3	*0.209*
TNM stage (I/II versus III)	72/10	38/15	*0.019*
Tumor size (> 3 cm versus ≤ 3 cm)	65/17	46/7	*0.264*
AFP (≥ 400 ng/ml versus < 400 ng/ml)	41/41	27/26	*0.915*
Tumor number (> 1 versus 1)	19/63	17/36	*0.253*
Vital status (Alive/Death)	68/14	20/33	*0.000*
HBsAg positive/negative	75/7	50/3	*0.533*

**Table 2:** Univariate and multivariate analyses of various prognostic parameters in patients with liver cancer by Cox-regression analysis

	Univariate analysis			Multivariate analysis		
	Relative risk	95% confidence interval	*P*	Relative risk	95% confidence interval	*P*
Tumor size	1.061	1.024–1.101	*0.001*	1.056	1.019–1.094	*0.002*
Tumor number	1.116	1.051–1.184	*0.000*	1.151	1.075–1.232	*0.000*
miR214	1.280	1.205–1.395	*0.000*	1.252	1.170–1.339	*0.000*
Child-Puge	1.562	1.322–1.847	*0.000*	1.219	1.013–1.466	*0.036*

**Table 3:** Spearman analysis of correlation between miR-214 and clinicopathological factors

Variables	miR-214 expression level	
	Spearman Correlation	*P* Value
Survival time	0.226	***0.008***
Vital status	−0.463	***0.000***
HBsAg	−0.054	*0.537*
Age	−0.1039	*0.230*
TNM	−0.203	*0.019*
AFP	0.018	*0.836*
gender	−0.108	*0.212*
Tumor number	0.029	*0.257*
Tumor size	0.072	*0.405*
Child-Pugh score	−0.087	*0.314*

### Overexpression of miR-214 inhibited cell proliferation and arrested cell cycle in HCC cells

**Figure 2:**
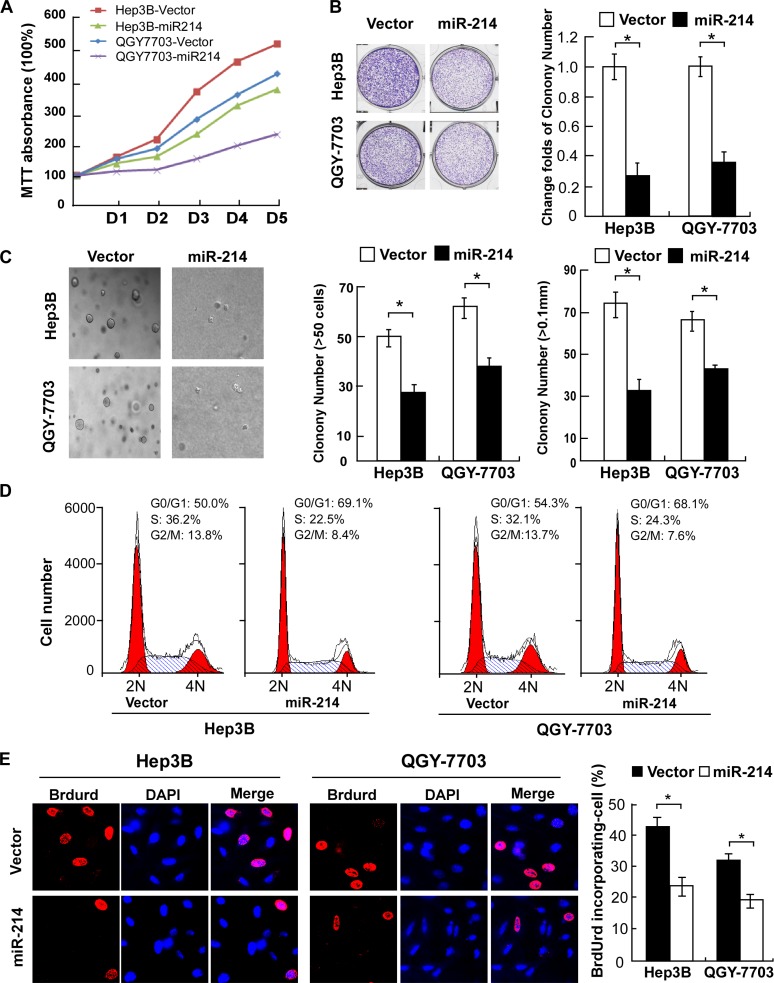
Overexpression of miR-214 suppressed HCC cell proliferation by inhibiting the G1–S transition **(A)** Growth curves of indicated cells in MTT assays. **(B)** Representative micrographs (left) and relative quantification (right) of crystal violet-stained cell colonies. **(C)** Representative micrographs (left) and relative quantification (right) of colonies between 25 and 50 mm in diameter for Hep3B cells or between 10 and 20 mm for QGY-7703 cells, counted in anchorage-independent growth assays. **(D)** Flow-cytometric determination of the proportion of indicated cells in distinct cell-cycle phases. **(E)** Representative micrographs (left) and quantification (right) of BrdUrd-incorporated cells in indicated engineered cell lines. *P < 0.05. DAPI, 4′,6-diamidino-2-phenylindole.

To explore the effect of miR-214 on cell growth, two HCC cell lines with moderate levels of miR-214 expression were forced to stably overexpress miR-214 to generate Hep3B-miR-214 and QGY-7703-miR-214 cell lines respectively. As shown in Figure 2A, an MTT cell viability assay indicted that overexpression of miR-214 significantly slowed down growth of both Hep3B and QGY-7703 cells compared with their corresponding controls. Furthermore, the colony forming capabilities of both cell lines were robustly suppressed by miR-214 transduction as compared with corresponding control cells (Figure 2B). Moreover, a soft agar assay showed that miR-214 overexpression dramatically attenuated anchorage-independent growth abilities of HCC cells, as evidenced by the number as well as the size of forming colonies measured (Figure 2C). Taken all together, these results suggested that overexpression of miR-214 suppressed the proliferation ability of HCC *in vitro*.

To understand the underlying mechanism of the alterations in cell proliferation caused by miR-214, fluorescence-activated cell sorting (FACS) was used to analyze the changes of DNA content throughout various phases of the cell cycle. As shown in Figure 2D, both miR-214–overexpressing Hep3B and QGY-7703 cells displayed a remarkable increase in the percentages of cells in G1-phase but decreased proportions of S-phase cells. Furthermore, consistent results from a BrdUrd incorporation assay confirmed that Hep3B-miR-214 and QGY-7703-miR-214 contained less BrdUrd-positive cells with newly synthesized DNA, 24.33% and 19.67%, respectively, than those in the control cell populations (43.67% and 32.33%, for Hep3B-vector and QGY-7703-vector cells, respectively, Figure 2E). Thus, our data suggests that miR-214 inhibits the G1–S transition of cell-cycle progression and consequently inhibited the proliferation of HCC cells.

### Antagonizing miR-214 accelerated HCC cell proliferation

**Figure 3:**
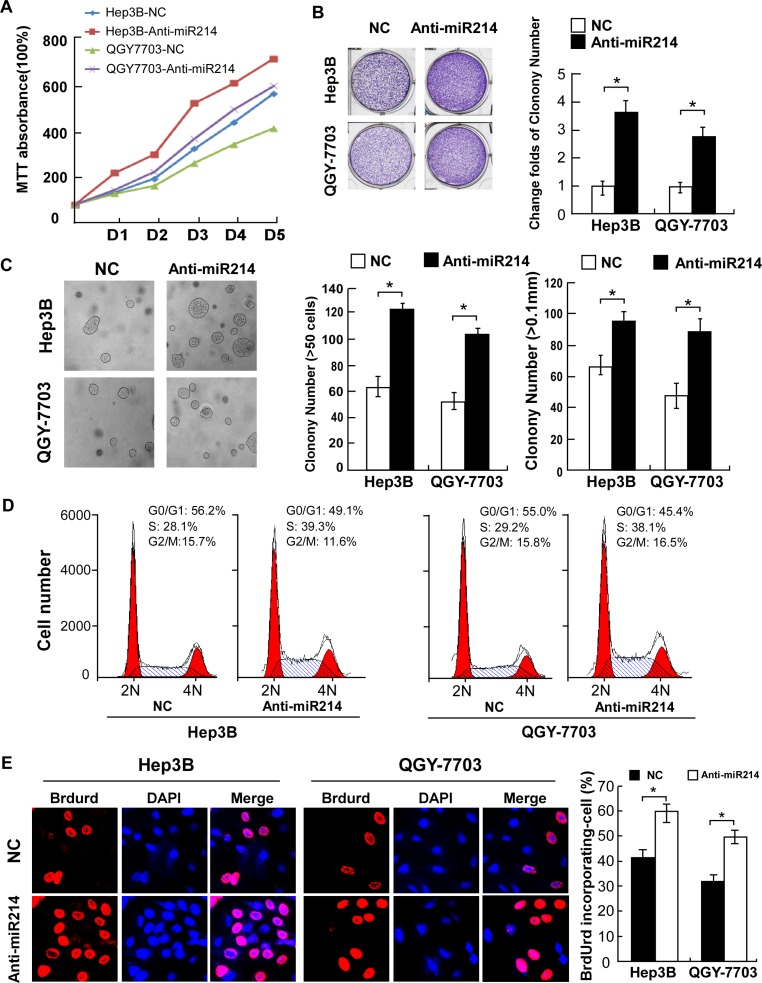
Antagonism of miR-214 accelerated proliferation of HCC cells by promoting cell-cycle progression **(A)** Growth curves of indicated cells in MTT assays. **(B)** Representative micrographs (left) and relative quantification (right) of crystal violet–stained cell colonies analyzed by colony formation assay. **(C)** Representative micrographs (left) and relative quantification (right) of colonies between 50 and 100 mm in diameter for Hep3B cells or between 20 and 50 mm for QGY-7703 cells. **(D)** Flow-cytometric analysis shows the proportion of indicated cells 48 hours after transfection with anti-miR or NC oligonucleotides. **(E)** Representative micrographs (left) and quantification of BrdUrd-incorporated cells in indicated cells. *P < 0.05.

To understand the role of endogenous miR-214 in the regulation of cell proliferation, anti-miR-214 oligonucleotides were used to silence endogenous miR-214 expression. As shown in Figure 3A, antagonizing miR-214 in Hep3B and QGY-7703 HCC cells drastically accelerated their proliferation as compared with their corresponding vector-control (NC) cells. Furthermore, colony formation and anchorage-independent growth assays revealed that after treatment with miR-214 antagonists, both Hep3B and QGY-7703 cells formed more and larger-sized colonies (Figure 3B and 3C). In parallel, antagonizing miR-214 significantly decreased the proportion of G1-phase HCC cells but increased the percentages of cells in the S-phase (Figure 3D). Immunofluorescence staining for BrdUrd incorporation further revealed elevated DNA synthesis by the miR-214 antagonists, showing 60.0% and 50.0% BrdUrd-positive cells in the Hep3B and the QGY-7703 lines, respectively, as compared with 41.67% and 32.33% for the corresponding vector-control cells (Figure 3E). Collectively, our antagonism experiments indicated that endogenous miR-214 might function as a cell-cycle suppressor, capable of abrogating the entry of HCC cells into the S-phase and thus suppressing cell proliferation.

### miR-214 suppresses expression of cell-cycle regulators CDK3, CDK6 and E2F2

**Figure 4:**
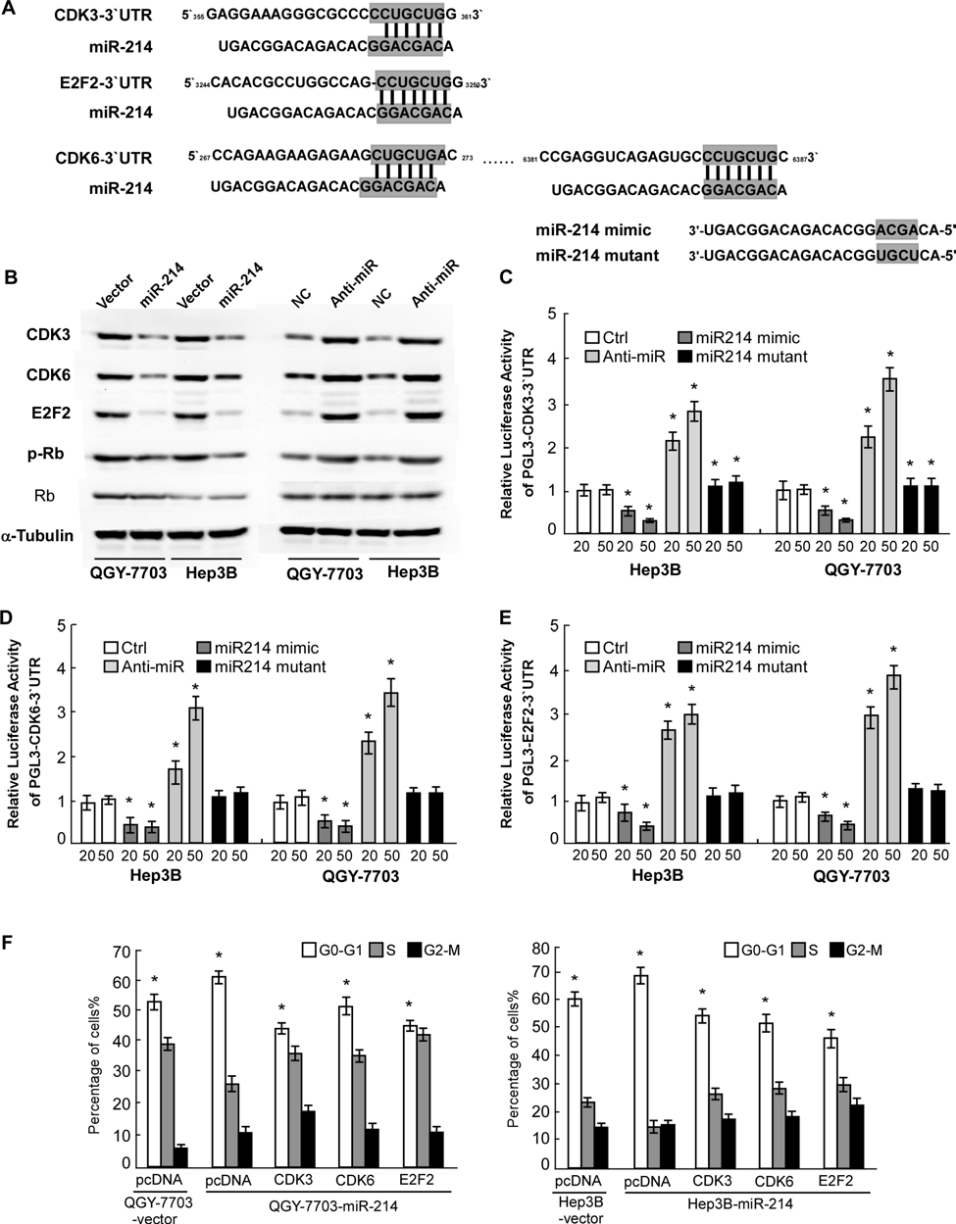
miR-214 directly inhibited E2F2, CDK3, and CDK6 cell-cycle regulators **(A)** Predicted binding of miR-214 to 3-UTRs of E2F2, CDK3, and CDK6. **(B)** Western blot showing E2F2, CDK3, and CDK6 expression in indicated cells. **(C)** Luciferase activity of pGL3-E2F2-3′-UTR, pGL3-CDK3-3′-UTR **(D)** or pGL3-CDK6-3′-UTR **(E)** reporter in indicated cells co-transfected with indicated oligonucleotides. **(F)** The effect of restoring ORFs (without 3-UTRs) of E2F2, CDK3, or CDK6 on proportions of indicated cells in distinct cell-cycle phases as determined by flow-cytometric analysis. For C to F, data are presented as mean ±SD from 3 independent experiments.

Next, using the DIANA-mirPath program, we investigated the mechanism by which miR-214/199a/199a* activates hepatitis B signaling, pathways in cancer and cell cycle, and found that 3 central cell-cycle promoting genes, E2F2, CDK3 and CDK6 was strikingly enriched in these predicted targets pathway. To elucidate the mechanism underlying the strong suppressive effect of miR-214 on HCC cell proliferation, we further evaluated the predicted target genes of miR-214 using Target Scan and studied the 3 genes (Figure 4A). As shown in Figure 4B, western blot confirmed that miR-214 mimics suppressed protein expression of E2F2, CDK3, and CDK6, while anti-miR-214 upregulated these genes protein expression, as compared with corresponding control cells. Consistently, phosphorylation of Rb was found to be decreased in miR-214–overexpressing cells but increased in the miR-214-inhibited cells. Furthermore, we found that miR-214 mimicking oligonucleotides dose-dependently abrogated the expression of luciferase activity of pGL3-E2F2, CDK3, CDK6-3′-UTR in HCC cells, and such suppressive effects could be reversed by anti-miR-214 oligonucleotides (Figure 4C–4E). However, mutating 4 nucleotides in the seed sequence of miR-214 completely abolished their binding to the target 3′-UTRs. Taken together, we concluded that miR-214 was able to directly bind the 3′-UTRs of E2F2, CDK3, and CDK6.

Based on the important role of E2F2, CDK3, and CDK6 in cell cycle progression, the effect of miR-214 on the cell-cycle was analyzed by flow cytometry. We reintroduced open reading frames (ORF) of the 3 genes into miR-214-overexpressing cells and assessed their cell-cycle distribution. The results showed that restoration of E2F2, CDK3, or CDK6 significantly rescued the G1-S transition impaired by miR-214 (Figure 4F), suggesting that downregulation of E2F2, CDK3, and CDK6 is functionally important for the inhibitory effect of miR-214 on HCC cell proliferation.

### Suppression of E2F2, CDK3, and CDK6 was functionally important for the biological effects of miR-214

**Figure 5:**
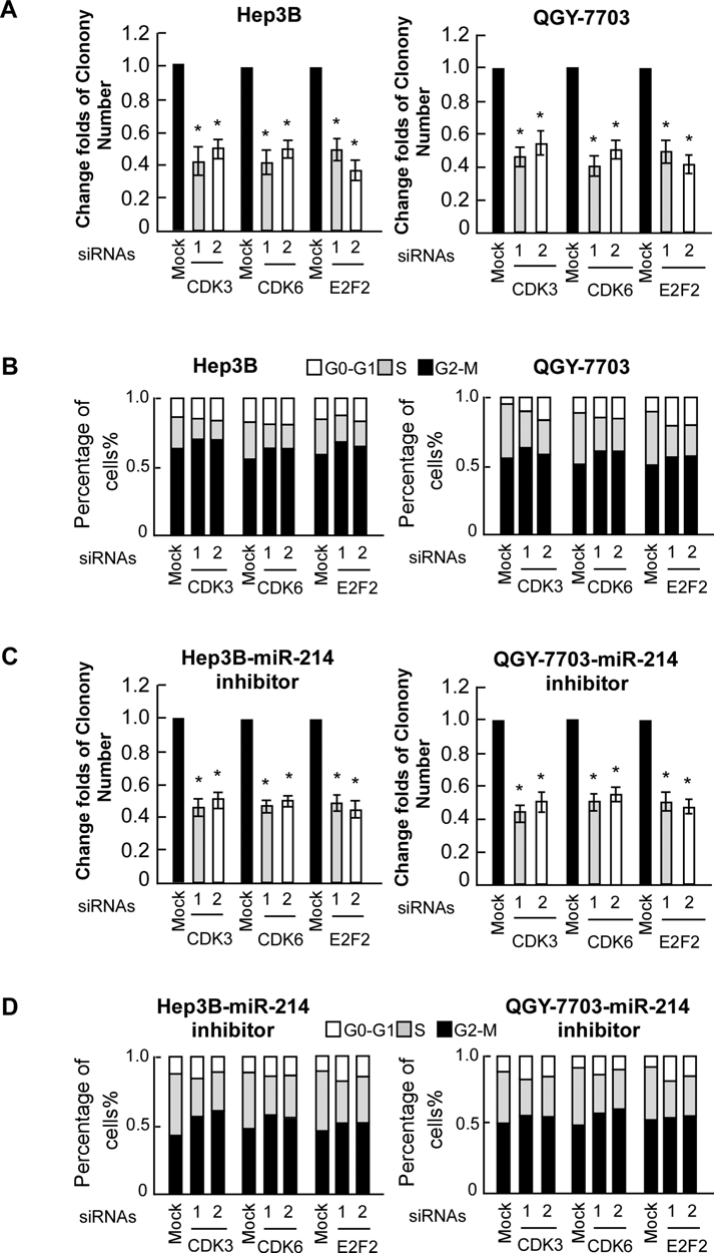
E2F2, CDK3, and CDK6 are functionally involved in miR-214–induced proliferation of HCC cell lines **(A)** Relative quantification of crystal violet–stained cell colonies of indicated cells. **(B)** Flow-cytometric determination of the proportion of indicated cells in distinct cell-cycle phases. **(C)** Relative quantification of crystal violet–stained cell colonies analyzed by clonogenic assays. Hep3B and QGY-7703 cells were treated with miR214 inhibitor and then transfected with indicated siRNAs. **(D)** Flow-cytometric determination of the proportion of indicated cells in distinct cell-cycle phases.

To explore the functional significance of E2F2, CDK3, and CDK6 in the proliferation of HCC cell lines and cell cycle activation induced by miR-214, we studied the effects of their depletion using specific siRNAs. As shown in Figure 5A, individually silencing E2F2, CDK3, and CDK6 in Hep3B and QGY-7703 cells led to decreased cell proliferation, as evidenced by anchorage-independent growth assay. Moreover, siRNA silencing of E2F2, CDK3, or CDK6 expression inhibited the cell entering the S stage (Figure 5B). In addition, the growth promoting effects resulting from mir-214 inactivation can be blunted by concomitant suppression of E2F2, CDK3, and CDK6 (Figure 5C and 5D). These results demonstrated that E2F2, CDK3, and CDK6 were functionally important for miR-214-induced cell proliferation in HCC cell lines.

### miR-214 inhibits HCC tumor growth *in vivo*

**Figure 6:**
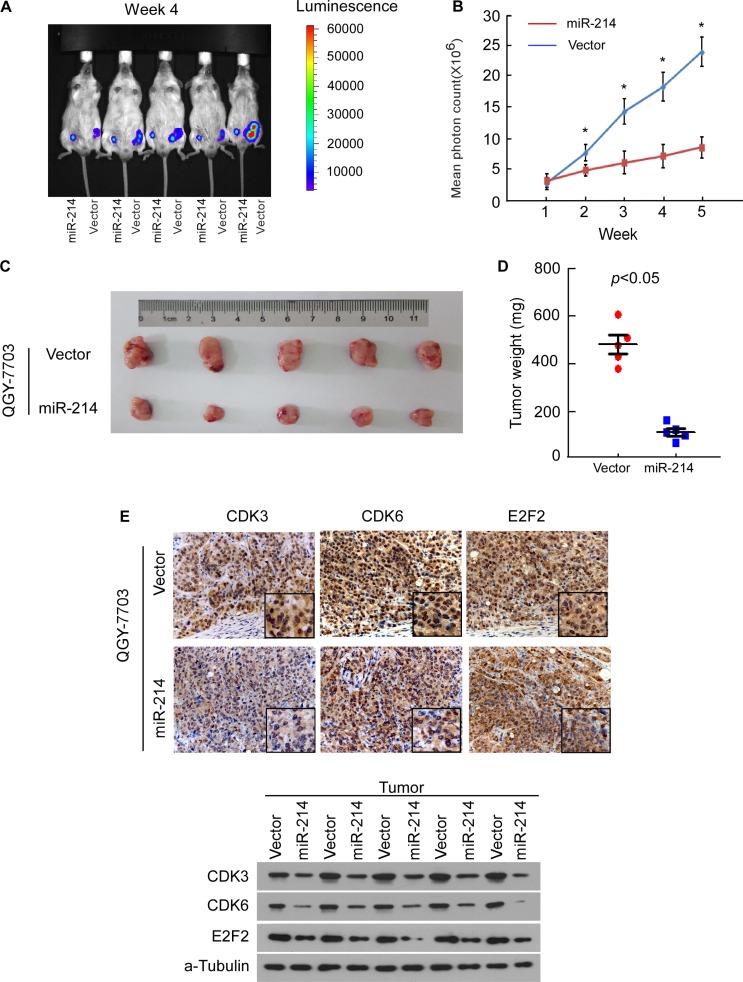
miR-214 inhibited tumor growth of HCC cell xenografts *in vivo* **(A)** QGY-7703/vector-luc cells and QGY-7703/miR-214-luc cells were injected into the groins of mice, and tumor growth of the indicated cells was monitored weekly by *in vivo* bioluminescence imaging. **(B)** Tumor bioluminescence signals were quantified by measuring mean photon radiance. **(C)** Representative volumes of tumor. **(D)** Representative graphs of tumor weights 5 weeks after inoculation. **(E)** IHC staining for E2F2, CDK3, and CDK6 in implanted tumors formed by indicated cells (top). Western blot confirmed protein expression of E2F2, CDK3, and CDK6 in indicated tumors (bottom). For B and D, data are presented as mean ± SD from 5 mice in each group, *P* < 0.05.

Considering the important role of miR-214 in HCC cell cycle and proliferation *in vitro*, we addressed the question of whether the miRNA acted as an endogenous antitumor factor *in vivo*. A bioluminescent imaging method was used to visualize and quantify the dynamic growth of subcutaneously xenografted liver tumors. Each experimental mouse was subcutaneously injected with miR-214-overexpressing or control HCC cells, on the right- or left-side dorsal thigh. By measuring the bioluminescence signal through quantifying the mean photon radiance, the miR-214-overexpressing QGY-7703 cells were found to exhibit a lower intensity of emanated bioluminescence than the control group cells after the first week, and the difference continued to be significant through the experimental endpoint (Figure 6A and 6B). The sizes and weights of dissected tumors at the end of experiment also confirmed the growth inhibitory effect of miR-214 (Figure 6C and 6D). Immunohistochemistry and Western blot also consistently showed that the expression of E2F2, CDK3, and CDK6 in miR-214-overexpressing cell-line tumor models were lower than those in the vector-control tumors (Figure 6E). These *in vivo* data strongly supported the notion that miR-214 functions biologically as a cell-cycle inhibitor and tumor suppressor, and that downregulation of miR-214 contributes to the development and progression of HCC tumors.

### Clinical relevance of miR-214 downregulation in human HCC

**Figure 7:**
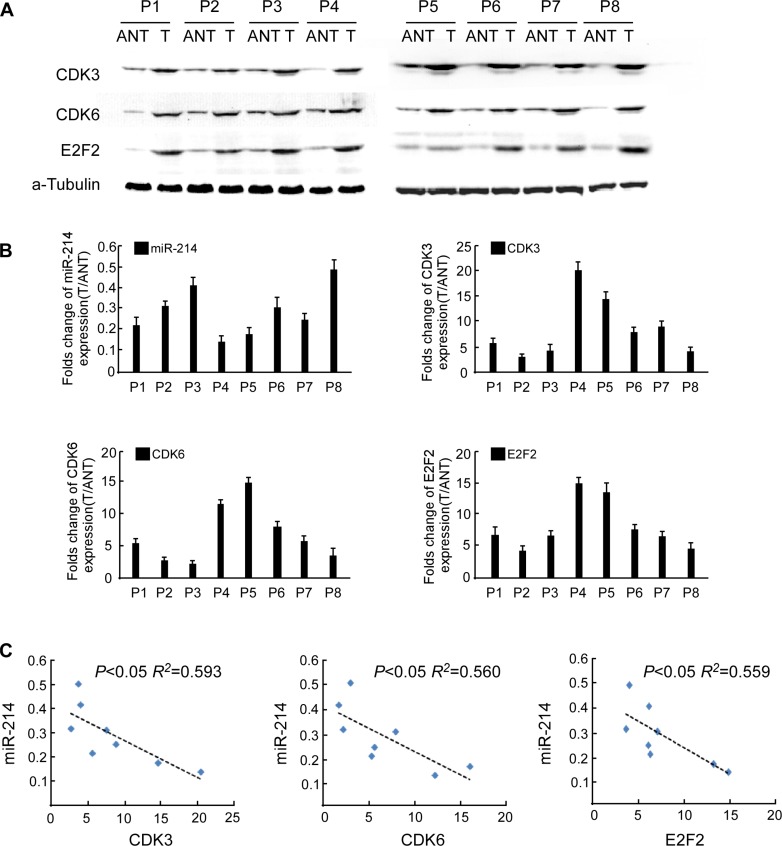
Clinical relevance of miR-214 expression and E2F2, CDK3, and CDK6 expression in human HCC tissues **(A)** Western blot of E2F2, CDK3, and CDK6 in 8 pairs of surgically resected fresh HCC tissues. **(B)** Fold change of miR-214 and mRNA levels of E2F2, CDK3, and CDK6 in HCC tissues (T) relative to adjacent noncancerous tissues (ANT) in each patient were quantified by RT-PCR and calculated. **(C)** Relationship of the expression of miR-214 and E2F2, CDK3, and CDK6 in HCC tissues.

To verify whether the biological effects of miR-214 on HCC cell proliferation were the same in clinical samples, we examined the correlation of miR-214 expression with the protein levels of E2F2, CDK3, and CDK6 in clinical specimens of HCC paired with adjacent noncancerous tissues. As shown in Figure 7A, significantly lower expression of miR-214 was found in the HCC lesions, in conjunction with elevated levels of E2F2, CDK3, and CDK6. This inverse correlation, as shown in Figure 7B, 7C, was quantitatively confirmed by RT-PCR. These data suggest that the inhibitory effect of miR-214 on its targets E2F2, CDK3, and CDK6, is clinically relevant in human HCC.

## DISCUSSION

The current study has demonstrated that miR-214 is a new tumor-suppressive miRNA in HCC that targets multiple cell-cycle stimulated gene (E2F2, CDK3, and CDK6) promoters. Our study proved that miR-214, as a member of the miR-214/199a/199a* cluster, had important effect on HCC prognosis. Experimental overexpression of miR-214 in HCC cells leads to suppression of E2F2, CDK3, and CDK6, cell-cycle arrest at the G1–S checkpoint, and disrupted proliferation of the cancer cells. Likewise, suppress miR-214 either alone or in association with E2F2, CDK3, and CDK6 in HCC cell lines, the growth promoting effects resulting from miR-214 inactivation can be blunted by concomitant suppression of E2F2, CDK3, and CDK6. Furthermore, our animal experiments and studies using clinical samples showed an inverse correlation between miR-214 levels and expression of E2F2, CDK3, and CDK6, as well as tumor progression. Notably, higher miR-214 expression, as well as the other members of the cluster miR-199a/199a*, is significantly associated with improved survival in patients with HCC, further suggesting a tumor-suppressive function of the molecule.

Decreased expression of miR-199a/199a* has been frequently demonstrated in HCC [13–15], and its decrement significantly correlates with the survival of HCC patients, outlining a potential marker for predicting the prognosis of HCC patients [16–18]. functional analysis and translational relevance of this phenomenon related to inhibition of cell proliferation, invasion by targeting the gene HIF-1α [19], E2F3 [20], DDR1 [21]. miR-214 was also found significantly reduced in HCC tissues consistent down-regulation of both miR-199a and miR-199a*. We wonder what is the role of miR-214 in the cluster in HCC. miR-214 has been shown to have tumor-suppressive effects in other human cancers. In chronic lymphocytic leukemia, miR-214 can negatively regulate the expression of PTEN [22]. In rhabdomyosarcoma, miR-214 regulatory loop suppresses cell growth and xenograft tumorigenesis [23]. Several previous studies reported its down-regulation in HCC as well. In particular, miR-214 down-regulation was associated with ER stress [24] and its enforced or inhibited expression was investigated in “*in vivo*” and “*in vitro*” models [25]. miR-214 deregulated expression was assessed as a contributor to tumor angiogenesis [26], invasion and early recurrence in HCC patients [27–29]. miR-214 was recently reported to promote hepatic fibrosis [30]. All the evidence proved miR-214 had a significant role in HCC metastasis and angiogenesis, however, the mechanism of cell cycle inhibition reported in these studies is not clearly identified. By conducting the current study, we have provided compelling biologic as well as clinical evidence that miR-214/199a/199a* expression is markedly downregulated in HCC, consistent with previous observations from profiling of miRNAs expression in HCC. And that miR-214 alone acts to suppress the proliferation of HCC cells both *in vitro* and *in vivo* through direct manipulation of cell cycle regulatory proteins. These results confirm the important role of miR-214 in the cluster as a tumor-suppressive miRNA in HCC. Thus, it remains important to thoroughly understand the molecular mechanisms mediating the differential biologic effects and targets of miR-214 in HCC and other cancer types.

HCC is frequently associated with abnormalities in cell cycle regulation. Our current study has identified three central cell-cycle regulators, namely, E2F2, CDK3, and CDK6, as targets of miR-214. These genes sequentially control cell-cycle checkpoints and thus have been described as proto-oncogenes and attractive therapeutic targets against cancer. E2F2, a member of the E2F family, activates the transcription of E2F target genes and stimulates the G1/S-phase transition [31–33]. Additional previous studies have provided evidence that E2F2 is upregulated in HCC and may be crucial in the promotion of cell proliferation [34–36]. CDK6 has been found to be amplified or overexpressed in glioma [37], lymphoma [38], and leukemia [39, 40], but little is known about CDK6 expression in HCC. CDK3, has been demonstrated to execute an essential function regulating S phase entry [41, 42]. It is very likely that E2F2, CDK6, and CDK3 act cooperatively to initiate or promote tumor development and progression. Therefore, silencing of all 3 genes could represent a more effective strategy of suppressing oncogenesis. Hence, identification of miR-214 as an efficient suppressor of E2F2, CDK3, and CDK6 provides new insights in our quest to develop more efficient approaches to treating HCC. On the other hand, the mechanism by which miR-214 is down-regulated in HCC remains uninvestigated. Since the main cause of HCC is HBV infection, it would be of great interest to further investigate whether miR-214 downregulation in HCC can be attributed to HBV inhibition with human hepatocytes.

In order to find novel therapeutic agents, it is important to identify effective biomarkers for the diagnosis and prognosis of HCC. To this end, our studies have found that miR-214 expression correlates with the clinical pathologic features and patient survival in HCC makes it a reasonable candidate biomarker for the determination of disease prognosis. Thus, these findings warrant further investigation into the potential development of miR-214-based prognostic and therapeutic approaches.

## MATERIALS AND METHODS

### Cell lines

Immortalized normal liver epithelial cells, THLE3, were maintained in bronchial epithelial growth medium (Lonza Cologne GmbH, Walkersville, MD), with 5 ng/ml epidermal growth factor, 70 ng/ml phosphoethanolamine and 10% FBS. HCC cell lines, including QGY-7701, QGY-7703, HCC-9810, SMMC-7721, Hep3B, PLC/PRF5, Hep3B, QGY-7703, Bel-7402, Bel-7404, MHCC97L, MHCC97H, HCCLM3 and HCCLM6, were grown in Dulbecco's modified Eagle's medium (DMEM) (Invitrogen, Carlsbad, CA) supplemented with 10% fetal bovine serum (FBS) (Invitrogen).

### Patient cohorts

Formalin-fixed, paraffin-embedded primary HCC specimens obtained from 135 patients, who underwent initial surgical resection between 2000 and 2005, were randomly selected from the archives of the Sun Yat-sen University Cancer Center (Guangzhou, China). Eight fresh HCC tissue samples, together with their paired adjacent non-cancerous tissues from each patient, were collected from HCC curative resection surgery. Cell free circulating miRNA was obtained by using HCC patients plasma that is collected in EDTA tubes. For the use of these clinical materials for research purposes, prior patients' consents and approval from the Institutional Research Ethics Committee of Sun Yat-Sen University Cancer Center were obtained. Detailed clinical information of all patients is summarized in Table 4.

**Table 4:** Clinicopathological characteristics of clinical samples and expression of miR-214/199a/199a* in liver cancer

Characteristics		No. Patients	%
Age (years)	≤ 50	85	63
	> 50	50	37
Gender	Male	122	90
	Female	13	10
TNM classification	I	12	8.9
	II	98	72.6
	III	25	18.5
HBsAg	Positive	125	92.6
	Negative	10	7.4
AFP	≥ 400 ng/ml	68	50.4
	< 400 ng/ml	67	49.6
Tumor size	> 3 cm	111	82.2
	≤ 3 cm	24	17.8
Tumor number	> 1	36	26.7
	= 1	99	73.3
Vital status (at follow-up)	Alive	88	65.2
	Death due to liver cancer	47	34.8
Expression of miR-214	High expression	83	61.5
	Low expression	52	38.5
Expression of miR-199a	High expression	80	59.3
	Low expression	55	40.7
Expression of miR-199a*	High expression	81	60
	Low expression	54	40

### RNA extraction, reverse transcription (RT) and real-time PCR

Total miRNA of experimental specimens was extracted using the mirVana miRNA Isolation Kit (Ambion). The expression levels of miR-214/199a/199a* were quantified with the miRNA-specific TaqMan miRNA Assay Kit (Applied Biosystems). U6 served as an internal control.

### Western blot

Western blot assay was proceeded as standard procedure. The membranes were incubated overnight at 4°C with anti-CDK3, anti-CDK6, anti-E2F2, anti-Rb or anti-p-Rb antibodies (BD PharMingen). Blotted membranes were blotted with an anti-a-Tubulin rabbit monoclonal antibody (Sigma) as a loading control.

### Xenograft tumor model *in vivo* and immunohistochemistry

Male BALB/c-nu mice (4–5 weeks of age, 15–18 g) were housed in barrier facilities on a 12 hours light/dark cycle. All experimental procedures were approved by the Institutional Animal Care and Use Committee of Sun Yat-Sen University. For the bioluminescent imaging assay, indicated cells (7 × 106, suspended in 100 mL sterile PBS, QGY7703-luc-Vector left and QGY7703-luc-miR-214 right) were injected subcutaneously into the flank of each dorsal thigh of BALB/C nude mice (*n* = 5). To estimate the tumor growth, bioluminescent imaging was also taken by using Xenogen IVIS Spectrum (Caliper Life Sciences). Fifteen minutes prior to imaging, mice were injected intraperitoneally with 150 mg/kg luciferin. Following general anesthesia, images were taken and analyzed with Spectrum Living Image 4.0 software (Caliper Life Sciences). After 5 weeks, mice were anesthetized and sacrificed, and tumors were removed, weighed, and sectioned, followed by real-time PCR analysis and immunohistochemical (IHC) analysis. After deparaffinization, sections were immunohistochemically analyzed using anti-CDK3, anti-CDK6, and anti-E2F2 antibodies (BD PharMingen). The resultant immunostaining images were captured using the AxioVision Rel.4.6 computerized image analysis system (Carl Zeiss).

### Plasmid and transfection

A DNA fragment containing the hsa-miR-214 precursor with 300 bp flanking sequence of each side was amplified into retroviral transfer plasmid pMSCV-puro (Clontech, Palo Alto, CA). Retroviral production and infection were performed as standard procedure. The open reading frames (ORFs) of CDK3, CDK6 and E2F2 generated by PCR amplification were cloned into the mammalian expression vector pcDNA3.1 (Invitrogen). 3′-UTRs of CDK3, CDK6 and E2F2 were amplified and then cloned into the downstream of the luciferase gene in a modified pGL3 control vector (Promega, Madison, WI). miR-214 mimics, anti-miR-214 oligonucleotides and their relative control oligonucleotides were purchased from Invitrogen. For depletion of genes, FlexiTube GeneSolution were used to knockdown for CDK3(GS1018, GIAGEN), CDK6(GS1021, GIAGEN), E2F2(GS1870, GIAGEN). QGY-7703/vector cells and QGY-7703/miR-214 HCC cells were infected with PMSCV-neo-luc2(QGY-7703/vector-luc cells and QGY-7703/miR-214-luc) to be tracked *in vivo* through bioluminescent imaging assay.

### 3-(4, 5-Dimethyl-2-thiazolyl)-2, 5-diphenyl-2H-tetrazolium bromide (MTT) assay

At indicated time points, cells seeded in 96-well plates were stained with 100 μl sterile MTT dye (0.5 mg/ml, Sigma) for 4 h at 37°C, followed by removal of the culture medium and addition of 150 μl of dimethyl sulphoxide (DMSO) (Sigma, St. Louis, MO, USA). The absorbance was measured at 570 nm, with 655 nm as the reference wavelength.

### Colony formation assay

Cells were plated on 6-well plates (0.5 × 103 cells for Hep3B and 2 × 103 cells for QGY-7703 per well) and cultured for 14 days. The colonies were stained with 1.0% crystal violet for 30s after fixation with 10% formaldehyde for 5 min.

### Anchorage-independent growth assay

Three thousand Hep3B cells and 1 × 10^4^ QGY-7703 cells were trypsinized and suspended in 2 ml complete medium plus 0.3% agar (Sigma, St Louis, MO). The agar-cell mixture was plated on the top of a bottom layer with 1% complete medium agar mixture. Following a 14-day incubation at 37°C, viable cell colonies with a diameter of 25–100 μm for Hep3B cells, or of 10–50 μm for QGY-7703 cells, were counted.

### Flow cytometry analysis

All cells in a culture dish were harvested and fixed in 80% ice-cold ethanol in PBS. Bovine pancreatic RNAase (Sigma-Aldrich) was added at a final concentration of 2 μg/ml, and cells were incubated at 37°C for 30 min, followed by incubation in 20 μg/ml of propidium iodide (Sigma-Aldrich) for 20 min at room temperature. Twenty thousand cells were analyzed on a flow cytometer (FACS Calibur; BD Biosciences).

### Bromodeoxyuridine labeling and immunofluorescence

Cells grown on coverslips (Fisher, Pittsburgh, PA) were incubated with bromodeoxyuridine (BrdUrd) for 1 h and stained with anti-BrdUrd antibody (Upstate, Temecula, CA). Gray level images were acquired under a fluorescence scanning microscope (Axioskop 2 plus, Carl Zeiss Co. Ltd., Jena, Germany).

### Luciferase assay

One hundred nanogram of pGL3-CDK3-3′UTR, pGL3-CDK6-3′UTR, pGL3-E2F2-3′UTR, plus 1ng of pRL-TK renilla plasmid (Promega, Madison, WI), were transfected using the Lipofectamine 2000 reagent (Invitrogen) according to the manufacturer's recommendation. Luciferase and renilla signals were measured at 48 h after transfection using the Dual Luciferase Reporter Assay Kit (Promega) according to a protocol provided by the manufacturer.

### Accession numbers

Microarray data described herein have been deposited in the Gene Expression Omnibus database (GEO GSE22058).

### Bioinformatics analysis

The following on-line software programs were used for bioinformatics analysis: TargetScan 4.1 (http://targetscan.org/vert_40/), DIANA-mirPath (http://diana.cslab.ece.ntua.gr/pathways/), and miRanda (http://www.microrna.org/microrna/getGeneForm.do). Bioinformatics analysis and visualization of microarray data were performed with the MeV v4.4 program (http://www.tm4.org/mev/).

### Statistical analysis

All HCC patients were divided into 2 groups based on miR-214/199a/199a* expression level, namely, the low or high expression group (less or more than the mean value), for clinical survival analysis. Statistical analyses were performed using the SPSS software program (SPSS Standard version 13.0). Comparisons between groups for statistical significance were conducted with a 2-tailed paired Student's *t*-test. The *X*^2^ test was used to analyze the relationship between miR-214 expression and clinicopathologic characteristics. Survival curve was plotted using the Kaplan–Meier method and compared by the log-rank test. *P* < 0.05 was considered statistically significant.
